# Perceptual, Semantic, and Pragmatic Factors Affect the Derivation of Contrastive Inferences

**DOI:** 10.1162/opmi_a_00165

**Published:** 2024-10-04

**Authors:** Camilo R. Ronderos, Helena Aparicio, Madeleine Long, Vishakha Shukla, Julian Jara-Ettinger, Paula Rubio-Fernandez

**Affiliations:** Department of Philosophy, University of Oslo, Oslo, Norway; Department of Linguistics, Cornell University, Ithaca, NY, USA; Department of Psychology, University of Edinburgh, Edinburgh, UK; Department of Communicative Sciences and Disorders, New York University, New York, NY, USA; Department of Psychology, Yale University, New Haven, CT, USA; Department of Computer Science, Yale University, New Haven, CT, USA; Max Planck Institute for Psycholinguistics, Nijmegen, Netherlands

**Keywords:** color adjectives, material adjectives, scalar adjectives, contrastive inference, pragmatics, visual salience

## Abstract

People derive contrastive inferences when interpreting adjectives (e.g., inferring that ‘the short pencil’ is being contrasted with a longer one). However, classic eye-tracking studies revealed contrastive inferences with scalar and material adjectives, but not with color adjectives. This was explained as a difference in listeners’ informativity expectations, since color adjectives are often used descriptively (hence not warranting a contrastive interpretation). Here we hypothesized that, beyond these pragmatic factors, perceptual factors (i.e., the relative perceptibility of color, material and scalar contrast) and semantic factors (i.e., the difference between gradable and non-gradable properties) also affect the real-time derivation of contrastive inferences. We tested these predictions in three languages with prenominal modification (English, Hindi, and Hungarian) and found that people derive contrastive inferences for color and scalar adjectives, but not for material adjectives. In addition, the processing of scalar adjectives was more context dependent than that of color and material adjectives, confirming that pragmatic, perceptual and semantic factors affect the derivation of contrastive inferences.

## INTRODUCTION

Understanding language requires more than decoding its literal meaning: pragmatic processes such as reference resolution (e.g., inferring who ‘John’ is in an utterance), disambiguation (e.g., identifying the intended meaning of ‘bank’) or implicature derivation (e.g., inferring that Sally did not eat all the cookies, if she ate ‘some cookies’) are pervasive in language comprehension. Here we investigated adjective interpretation with a focus on *contrastive inference*: adjectives are interpreted contrastively when they are taken to distinguish the intended referent from other entities of the same kind, rather than being merely descriptive. For example, in processing ‘the short pencil,’ a listener would normally understand that the speaker is contrasting a short pencil with a longer one, and the derivation of this pragmatic meaning is a contrastive inference. Our study tested the hypothesis that not only pragmatic but also semantic and perceptual factors affect the derivation of contrastive inferences during language processing.

Using an elegant eye-tracking paradigm that is now a classic in psycholinguistics, Sedivy et al. (1999; Sedivy, 2003, 2004) investigated the derivation of contrastive inferences during real-time language comprehension. Numerous studies have since employed this paradigm to study adjective interpretation under different conditions, both online (e.g., Grodner & Sedivy, 2011; Ryskin et al., 2019) and offline (Kreiss & Degen, 2020). However, recent eye-tracking studies have only partially replicated the original results with different types of adjectives (Aparicio, 2017; Aparicio et al., 2015; Rubio-Fernandez et al., 2021; Ryskin et al., 2023; Saryazdi et al., 2022; for details, see below). In addition, it has been recently shown that the derivation of contrastive inferences varies across languages depending on adjective position (i.e., prenominal vs postnominal; Rubio-Fernandez & Jara-Ettinger, 2020). These results call for a closer investigation of how contrastive inferences are derived online; in particular, which factors influence the derivation of these inferences and whether their derivation can be generalized across different languages with prenominal adjectival modification.

The main aim of this study is therefore to investigate the interpretation of three different types of adjectives—namely, color (e.g., ‘the black lamp’), material (e.g., ‘the leather shoes’) and scalar (e.g., ‘the short pencil’), in three languages with prenominal modification—namely, English, Hindi, and Hungarian. More specifically, we used Sedivy’s classic eye-tracking paradigm to explore how pragmatic, perceptual and semantic factors may affect the derivation of contrastive inferences in languages with prenominal adjectives. In addressing this research question, our study aims to contribute to a better understanding of the mechanisms behind the derivation of contrastive inferences, which can in turn help explain the discrepancies observed in previous studies.

In what follows, we will first introduce Sedivy’s original account of contrastive inference as a pragmatic phenomenon. We will review the empirical evidence supporting this account, as well as more recent findings challenging its strongest conclusions (Pragmatic Factors in the Derivation of Contrastive Inferences section). Given the relatively limited explanatory power of the pragmatic account, we will discuss perceptual factors that might also affect the derivation of contrastive inferences during adjective interpretation. More specifically, we will review recent findings that color is more visually salient than material (Jara-Ettinger & Rubio-Fernandez, 2022; Kursat & Degen, 2021) and discuss the role that visual salience plays in Sedivy’s eye-tracking paradigm (Perceptual Factors in the Derivation of Contrastive Inferences section). Finally, we will consider semantic factors potentially affecting the derivation of contrastive inferences; in particular, the difference between scalar adjectives, on the one hand, and color and material, on the other (Semantic Factors in the Derivation of Contrastive Inferences section). We will then report our cross-linguistic eye-tracking experiment (Methods section) and discuss our findings (Discussion section).

### Pragmatic Factors in the Derivation of Contrastive Inferences

**Figure 1.**
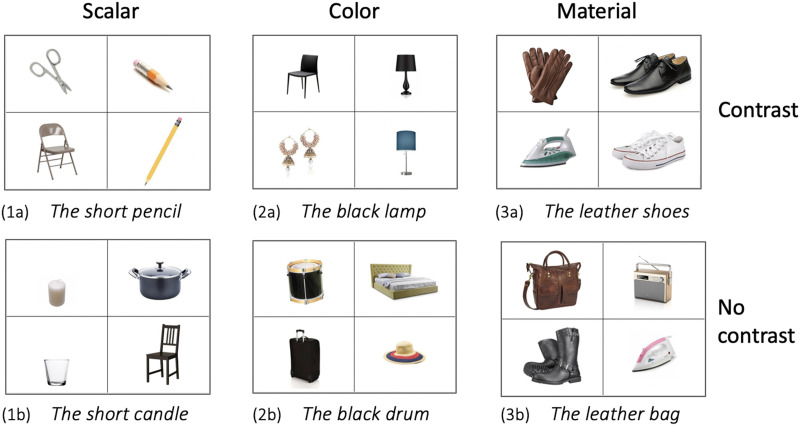
Sample displays from the three adjective types in the two experimental conditions. The description of the target (which was presented auditorily in English, Hindi, and Hungarian) is written under each display.

In the first paper to use this paradigm, Sedivy et al. (1999) observed that participants derived contrastive inferences when interpreting scalar adjectives. Participants had to move objects in a display following descriptions such as ‘the short pencil’, while their eyes were being tracked. Critical trials had a *contrast object* (e.g., a longer pencil), supporting a contrastive interpretation of the adjective, whereas control trials did not, only allowing for a descriptive interpretation of the adjective (see Figure 1(1a–1b)). Contrastive interpretation was measured by comparing fixations on the *target object* relative to fixations to the *competitor object* (e.g., short scissors), which did not have a contrast in the display. Thus, when processing ‘Pick up the short …’ in display (1a), participants who derive a contrastive inference should anticipate the target before hearing the noun. That is, in hearing ‘short’, participants should fixate on the short pencil (because there is a long pencil in the display) and not on the short scissors (because there are no long scissors). However, in the no-contrast condition in (1b), the temporary ambiguity created by ‘short’ could only be resolved when hearing the noun, with participants fixating comparably on the target and the competitor objects while processing the adjective.

In a follow-up study, Sedivy (2004) replicated these results with scalar and material adjectives, but not with color adjectives. This was interpreted as an effect of the varying frequency with which these adjectives were used descriptively in a language production task: participants produced color adjectives in descriptive, non-contrastive contexts around half the time, but rarely did so with material and scalar adjectives. Sedivy (2003, 2004) argued that the derivation of contrastive inferences is driven by *informativity expectations* (Grice, 1975): when a speaker produces a material or scalar adjective, the listener is entitled to assume that the adjective has a contrastive function because that is how those adjectives are most commonly used. However, this expectation is not warranted when a speaker produces a color adjective, because those are also used in non-contrastive contexts.

Supporting the informativity account of contrastive inferences, Sedivy (2003) showed that, when describing objects with predictable colors (e.g., a yellow banana), speakers only used color adjectives contrastively (e.g., if there was also a green banana in the display). Moreover, listeners were faster to identify ‘the yellow banana’ when a green banana made the color adjective contrastive, relative to when the predictable color was purely descriptive. Sedivy (2003) concluded that contrastive inferences are triggered by listeners’ informativity expectations and are therefore pragmatic in nature.

Sedivy’s (2003, 2004) results support the pragmatic view of contrastive inferences, but her account faces some challenges. First, participants in her language production task used descriptive color adjectives less than half of the time when naming objects with unpredictable colors (Sedivy, 2004), making it unlikely that listeners expect color words to be used descriptively. Moreover, recent referential communication studies have shown that speakers’ tendency to use color adjectives descriptively varies greatly depending on the discriminability of the referent (Koolen et al., 2013; Long et al., 2021; Rubio-Fernandez, 2021), the density of the display (Clarke et al., 2013; Gatt et al., 2017; Rubio-Fernandez, 2019), the lexical category of the noun (Rubio-Fernandez, 2016, 2019; see also Long & Rubio-Fernandez, 2024; Rohde & Rubio-Fernandez, 2022) and the position of the adjective (Rubio-Fernandez, 2016, 2019; Wu & Gibson, 2021). The variability observed in these studies has been interpreted as an effect of the varying efficiency of descriptive color modification in different visual contexts (Rubio-Fernandez et al., 2021), challenging the view that color adjectives are part of the ‘default description of objects’ (Sedivy, 2003, 2004).

Moreover, recent eye-tracking studies using Sedivy’s paradigm have observed contrastive effects with color adjectives (Aparicio, 2017; Aparicio et al., 2015; Ryskin et al., 2023; Saryazdi et al., 2022), contrary to the original findings. A methodological difference that might explain these different results is that Sedivy (2003, 2004) used a *target-advantage measure*, calculated as the proportion of time participants spent looking at the target minus the proportion of time spent looking at the competitor, whereas Aparicio et al. (2015; Aparicio, 2017) compared fixations to the target object across the two experimental conditions. However, Saryazdi et al. (2022) recently observed contrastive effects with color adjectives using the original target-advantage measure, and Ryskin et al. (2023) observed contrastive effects with color and size adjectives using both measures, which suggests that the discrepant findings are not due to the different eye-tracking measures employed in these studies.

### Perceptual Factors in the Derivation of Contrastive Inferences

According to Sedivy (2004), listeners have different informativity expectations for color and material adjectives because color adjectives are often used descriptively. Therefore, from a pragmatics perspective, we should expect greater contrastive effects for material than for color adjectives, as Sedivy (2004) did observe. However, another important difference between color and material adjectives is the relative visual salience of the properties they encode, with color being normally more salient than material. This hypothesis was recently tested in an eye-tracking study comparing target identification in contrastive displays following color versus material descriptions of the targets (Jara-Ettinger & Rubio-Fernandez, 2022). Eye-movements and response times revealed that target identification was faster in the color condition than in the material condition. For example, when presented with a display of four objects, including a red leather chair and a green plastic chair, participants fixated longer on the target object and less on the contrast object when the target was described as ‘the red chair’ than when it was described as ‘the leather chair’. These different looking patterns resulted in shorter response times in the color condition than in the material condition.

Further empirical evidence that color is more visually salient than material comes from Kursat and Degen (2021), who conducted two separate norming experiments investigating the relative perceptual difficulty of color and material properties. Participants were presented with a series 81 object images paired with a color or a material adjective and they were under time pressure to confirm (or disconfirm) whether each object had the property in question. In both experiments, material adjectives resulted in higher error rates and longer response times than color adjectives, confirming that detecting an object’s material is more difficult than detecting its color.

The results of Jara-Ettinger and Rubio-Fernandez’s (2022) eye-tracking study and Kursat and Degen’s (2021) norming experiments suggest that, from a perceptual perspective, color adjectives would be expected to elicit greater contrast effects than material adjectives. This prediction rests on the assumption that in Sedivy’s eye-tracking paradigm, participants must conceptualize the two objects of the same kind as *contrasting along a certain dimension* (e.g., two pencils of different lengths or two lamps of different colors, as in Figure 1) in order to be able to anticipate the noun when processing the adjective. Thus, during the preview of display (2a), participants should readily perceive the color contrast between the two lamps, which would prepare them to anticipate the noun when hearing ‘the black …’. Material adjectives, on the other hand, do not encode equally salient visual properties (Jara-Ettinger & Rubio-Fernandez, 2022; Kursat & Degen, 2021) and therefore, during the preview of display (3a), the two pairs of shoes may not be conceptualized as contrasting in material, preventing participants from anticipating the noun ‘shoes’ when hearing ‘the leather …’.

Pragmatic and perceptual analyses of different adjective types therefore predict different processing blueprints for color and material adjectives, which we tested here using Sedivy’s classic paradigm.

### Semantic Factors in the Derivation of Contrastive Inferences

Color, material and scalar adjectives not only differ in the informativity expectations they create in language processing—given how they are used in language production (Sedivy, 2003, 2004) and in their visual salience (Jara-Ettinger & Rubio-Fernandez, 2022; Kursat & Degen, 2021) but also in their semantics: scalar adjectives are interpreted in relation to a *comparison class*, whereas color and material adjectives are interpreted in absolute terms because they encode non-gradable properties. For example, a pencil may be short compared to a longer pencil, or in relation to the average length of pencils, but it can be yellow or wooden in and of itself (Kennedy, 1999, 2007; Kennedy & McNally, 2005). Recent findings suggest that adjective semantics might also play a role in the derivation of contrastive inferences.

Aparicio et al. (2015; Aparicio, 2017) compared the processing of color and scalar adjectives using Sedivy’s eye-tracking paradigm. They employed the No-Contrast condition as a baseline to establish how quickly participants can identify different types of adjectival properties in a visual display and observed that participants were faster to attribute color than scalar properties (i.e., participants identified the two color-matching objects faster than the two scalar-matching objects). Aparicio and colleagues argued that these different looking patterns may result from the different semantics of these adjectives, with scalar adjectives being more context dependent than color adjectives, possibly delaying lexical processing in the absence of contrast. Aparicio et al. (2015; Aparicio, 2017) also considered the possibility that color-matching objects might receive more fixations than scalar-matching objects because color is more visually salient than scalar properties—a hypothesis we further investigated in our study.

The processing of color and size adjectives were recently compared in another eye-tracking study using Sedivy’s paradigm with native speakers of Tsimane’ and English, who fixated more on the target in color trials than in size trials, overall (Ryskin et al., 2023). This looking pattern is in line with Aparicio et al.’s (2015) semantic analysis of scalar versus color adjectives. To further test this analysis, here we compared overall looks to the target and the competitor in the No-Contrast condition when processing scalar versus color adjectives, and extended that comparison to scalar versus material adjectives. As Aparicio et al. (2015; Aparicio, 2017) acknowledge, because color is so visually salient, it is possible that greater looks to color-matching objects than to scalar-matching objects might result from this perceptual factor, and not only from the different semantics of these adjectives. Therefore, a comparison between scalar and material adjectives offers a better test case for a semantic effect, since material adjectives encode a non-gradable property that is not as visually salient (Jara-Ettinger & Rubio-Fernandez, 2022; Kursat & Degen, 2021).

In summary, the present study aims to tease apart the effect of pragmatic and perceptual factors in the derivation of contrastive inferences by testing different predictions for color and material adjectives. In addition, we will test the effect of semantic factors in the processing of scalar adjectives by comparing them to color and material adjectives in the baseline condition. It must be noted that whereas all three factors (i.e., pragmatic, perceptual and semantic) may have additive effects in the derivation of contrastive inferences, the present study was designed to isolate these different effects, rather than testing their combined effects. Finally, a recent eye-tracking study using Sedivy’s paradigm revealed that the derivation of contrastive inferences facilitates adjective processing only in languages with prenominal modification (Rubio-Fernandez & Jara-Ettinger, 2020). It follows from this cross-linguistic study that under the same experimental conditions, pragmatic, perceptual and semantic factors should similarly affect the derivation of contrastive inferences in different languages with prenominal modification. Therefore, for a more robust test of our predictions, we ran the same eye-tracking task with native speakers of English, Hindi, and Hungarian.

## METHODS

### Participants

108 participants took part in the experiment. 60 native English speakers were recruited from the participant pool at the Department of Brain and Cognitive Sciences at MIT. Of these, 11 had to be excluded because of a malfunction of the mouse connected to the eye-tracker, leaving the total at 49.

Another two language groups were recruited to perform the same eye-tracking task for a study that has already been published (Rubio-Fernandez & Jara-Ettinger, 2020): 27 native speakers of Hindi from the Indian Institute of Technology in Delhi, and 21 native speakers of Hungarian from the Central European University in Budapest.^1^

The total number of participants for which data was analyzed in the present study was 97. All participants received monetary compensation for their time.

### Materials, Design and Procedure

A list of 8 English color adjectives (black, blue, brown, green, orange, red, white and yellow), 8 material adjectives (cotton, glass, gold, leather, metal, paper, plastic, wooden and woolen) and 8 scalar adjectives (large, narrow, short, small, tall, thick, thin and wide) were compiled for the study. These were then translated to Hindi and Hungarian by native speakers.

The experiment had a 2 × 3 block design, with the factor CONDITION (Contrast vs No-Contrast) administered in two separate trial blocks and the factor ADJECTIVE TYPE (Scalar, Material and Color) administered across both trial blocks. Two displays of four objects were created for each adjective, one for each level of the factor CONDITION. Participants therefore saw 8 trials per ADJECTIVE TYPE in each trial block (see Figure 1). In the No-Contrast condition, the display included an object that shared the relevant property with the target, whereas in the Contrast condition, the display included both an object that shared the relevant property with the target and an object of the same kind as the target (i.e., the competitor). Target and competitor objects differed along various dimensions (e.g., in the Material and Scalar conditions, target and competitor differed not only by these properties but also by color, and in the Color condition, target and competitor differed by shape and material). The remaining object(s) in the experimental displays were of a different kind and did not share the relevant property with the target object and were therefore treated as distractors. English sample items are shown in Figure 1. A set of 4 displays were used as warm-up trials and another set of 48 displays (24 with competitors and 24 without competitors) were used as fillers. Warm-up and filler trials were not analyzed.

Target descriptions were recorded by a native speaker of each of the three languages. None of them stressed the adjectives contrastively. Unlike in previous studies using this paradigm, target descriptions were not embedded in a full instruction (i.e., participants heard ‘the short pencil’ rather than ‘pick up the short pencil’ or ‘click on the short pencil’) in order to avoid syntactic differences across the languages.

The materials were presented in two blocks of trials, with a 10 second break in between the two blocks. In the first block, all displays contained two objects of the same kind and were comprised of the 24 trials from the Contrast condition plus 24 fillers. The target was one of the two objects of the same kind in the experimental condition and one of the other two objects in the filler trials. The target description included an adjective in the Contrast condition, but not in the filler trials (e.g., ‘the short pencil’ was used in a display with two pencils and ‘the elephant’ in a display with a single elephant). The filler trials were designed to prevent participants from anticipating that the target was one of the two objects of the same kind in critical trials (since that was not the case in the filler trials).

In the second block of trials, all four items in each display were different types of objects, but two of the objects shared a property. The target was always described using a descriptive adjective. As in the first block, the filler trials were designed to prevent participants from anticipating that the target was one of the two objects that shared a property in critical trials (since that was not the case in the filler trials).

The 24 adjectives selected for the study were used once in the first block of trials and twice in the second because the same adjectives were used in the filler trials. However, the 24 adjectives modified 72 different nouns in order to prevent participants from anticipating the target when the adjectives were repeated in the second block of trials. Trial presentation was randomized individually within each block.

This block design was intended to prevent participants from lowering their expectations of contrastive modification if descriptive trials from the No-Contrast condition were intermixed with critical trials from the Contrast condition (as previously observed by Grodner & Sedivy, 2011; Ryskin et al., 2019).

Participants had 3 seconds to preview the display before the description of the target started. The preview was relatively long to allow participants to compare the objects and conceptualize the relevant contrast prior to hearing the utterance. From the onset of the target description, participants had 3 seconds to click on the target. In between trials, participants had to click on a cross in the center of the screen to ensure that the cursor was equidistant from the four objects at the start of each trial. Participants were asked to inspect the display during the preview window and click on the target object as fast and accurately as they could when hearing its description. Participants could therefore click on the target before the end of the description, with trial duration being set to 3 seconds in the two blocks.

Eye movements from all participants were recorded with a portable eye-tracking system (RED-m by SMI) that measured eye position at a sampling rate of 120 Hz and had a spatial resolution (RMS) of 0.1° and an accuracy of 0.5°. Participants were seated about 60 cm from the computer screen, with a contact-free set up. The task lasted approximately 20 min.

### Analyses

#### Preprocessing.

For data pre-processing, visualization and analysis we used the R programming language (R Core Team, 2020) and RStudio (RStudio Team, 2020). We also used the following R packages: lme4 (Bates et al., 2007), Rmisc (Hope, 2013), MASS (Ripley et al., 2013), eyetrackingR (Dink & Ferguson, 2015), the Tidyverse suite of packages (Wickham et al., 2019) and afex (Singmann et al., 2015). Data and code necessary to replicate our analyses and data visualizations can be found on the project’s OSF repository: https://osf.io/apxtj/?view_only=285b127c526b4ef58491ae3d963d3735.

Prior to data analysis, we computed the proportions of fixations on the target image for every 20-millisecond time bin. This was done time-locked from onset to offset of the noun (offset by 200 milliseconds to account for the time it takes to plan and execute a saccade, as is typically done in Visual-World experiments). We chose this region for two reasons: First, in our experiment, participants heard the definite description (e.g., ‘the black lamp’) at a normal speech rate without prior sentential information (e.g, ‘Pick up …’ or ‘Click on …’). This is likely to delay the earliest anticipatory effects that could arise using this paradigm. Second and more importantly, the duration of the adjectives varied between languages and between adjective types. The average adjective length in English was the longest (color: 533 ms., material: 904 ms., scalar: 606 ms.) followed by Hungarian (color: 530 ms., material: 468 ms., scalar: 536 ms.), and Hindi (color: 411 ms., material: 490 ms., scalar: 432 ms.). This difference made it so that, across languages and adjective types, we could only be certain that every participant had understood each adjective by taking the noun onset as the starting point of the analysis.

#### Cluster-Based Permutation.

To analyze the data, we took two different approaches. First, we performed a *cluster-based permutation analysis* (Maris & Oostenveld, 2007; for a recent application of this method in pragmatics, see Huang & Snedeker, 2020). The cluster-based permutation analysis is a non-parametric test which helps determine whether a difference between conditions is significant somewhere within a pre-determined temporal region of interest. This analysis considers the temporal dynamics of the experiment by performing a separate test on each time-bin. However, it does not inflate the false positive rate because inferences regarding the differences between conditions are drawn based on a single significance test performed at the end (against a bootstrapped null-hypothesis).

The analysis itself involves various steps. The first step is to fit a model to every time-bin within the region. We did this for each adjective type individually (i.e., to check for an effect of CONDITION for each level of the factor ADJECTIVE TYPE), and then for the interaction effect of the two factors. For each comparison, we fitted a mixed-effects logistic regression model. The models for each adjective included CONDITION as a fixed effect as well as random intercepts and slopes for CONDITION by participants and languages and random intercepts by items. The models for the interaction included the CONDITION * ADJECTIVE TYPE interaction as a main effect (with CONDITION sum-contrast coded and ADJECTIVE TYPE helmert-contrast coded, isolating the interaction effect between material adjectives, on the one hand, and color and scalar adjectives, on the other), and as a random effect by participants and languages, with random intercepts by items, participants, and languages. Since the visual display of each item varied between conditions, this was the ‘maximal’ random-effects structure for all models (see Barr et al., 2013).

Note that we did not include LANGUAGE as a predictor in the models because we were not interested in the differences between individual languages, but in whether contrastive inference effects can be generalized across languages with prenominal modification. For this reason, LANGUAGE was included as a clustering unit in the random effects structure that is superordinate to participants (since each participant appears in one language group only). This means that our models consider the random variation between participant nested within the random variation by language. In R syntax, this nesting of clustering units is expressed with a forward slash as (1 + *AdjectiveType*‖*Language*/*Participant*).

The second step was to establish clusters of significant effects. This was done by counting the number of contiguous time-bins displaying a significant effect going in the same direction. The next step involved determining the probability that any cluster of effects found could arise by chance. To do this, a non-parametric permutation test was performed on the same data 1000 times, in which the condition labels were randomly switched within participants, creating a bootstrapped null distribution. In the final step, a p-value was extracted for each comparison (i.e., for the effect of CONDITION on each adjective type and for the interaction of the two factors) by counting the number of times (out of the 1000 permutation tests) in which a cluster of effects of the size found (or bigger) was present in the null-distribution with the permuted labels (see Ito and Knoeferle (2023) for a review of this method and a comparison to other methods used with the Visual-World Paradigm).

#### Target-Advantage Scores.

Our second approach to data analysis was to compute a target-advantage score for the entire region, as was done by Sedivy (2004), for the three different adjective types (see also Ryskin et al., 2023; Saryazdi et al., 2022). This was done to make our results more comparable to the previous literature. To compute this score, we calculated the amount of time participants spent fixating on the target and on the competitor images throughout the critical time-window in each trial (relative to the total duration of each critical time-window). We then subtracted the proportion of time spent viewing the competitor from the time spent viewing the target. We fitted a ‘maximal’ mixed-effects linear regression model to this resulting target advantage score. The model included both factors and their interaction as fixed effects. It also included random slopes for both effects and their interaction by participants and languages, as well as random intercepts by participants, languages and items. The model used a sum-contrast coding scheme for the factor CONDITION, and a treatment contrast-coding scheme for the factor ADJECTIVE TYPE. Three versions of this model were run, changing the condition coded as the intercept each time in order to test the main effect of CONDITION on each level of the factor ADJECTIVE TYPE. P-values were computed for the regression models using the Satterthwaite approximation.

As a control measure, we also analyzed the target advantage scores across conditions prior to the onset of the noun region (from the beginning of the trial until the noun onset; mean = 540 ms). We did this to rule out the possibility that, given our block design, participants’ gaze behavior in the Contrast condition might have been caused by their developing a block-specific strategy that allowed them to anticipate the target image irrespective of the heard adjective. Such a strategy would translate to more looks to the target (and less to the competitor) in the Contrast versus No-Contrast condition prior to the critical noun (i.e., a baseline advantage). We thus fitted a mixed-effects regression model to the target advantage scores in the time prior to hearing the critical noun. The model had random intercepts and slopes by participant and language and random intercepts by items. The fixed effects were CONDITION and ADJECTIVE TYPE (both sum-contrast coded), plus their interaction.

#### Total Number of Looks.

As a final measure, we analyzed the total number of looks across both target and competitor images in the No-Contrast condition. We did this to test the hypothesis that scalar adjectives might require additional processing when compared to color and material adjectives because their interpretation requires an evaluation of the relevant comparison class (Aparicio, 2017; Aparicio et al., 2015). This is likely to result in a delayed identification of the scalar-matching objects in the visual display, since participants have to more closely examine *all* of the available images to determine the comparison class. Color and material adjectives, on the other hand, do not require this comparison. We followed Aparicio et al. (2015) in focusing on the No-Contrast condition because it can provide a baseline measure of how people understand different types of adjectival properties when the visual context does not give rise to a contrastive inference. To test this hypothesis, we fitted another ‘maximal’ mixed-effects logistic regression model on the overall proportion of looks across the noun time-window for the No-Contrast condition, using ADJECTIVE TYPE as a sole predictor. ADJECTIVE TYPE was treatment-contrast coded, with scalar adjectives coded as the intercept of the model in order to compare it to color and material adjectives directly.

## RESULTS

**Figure 2.**
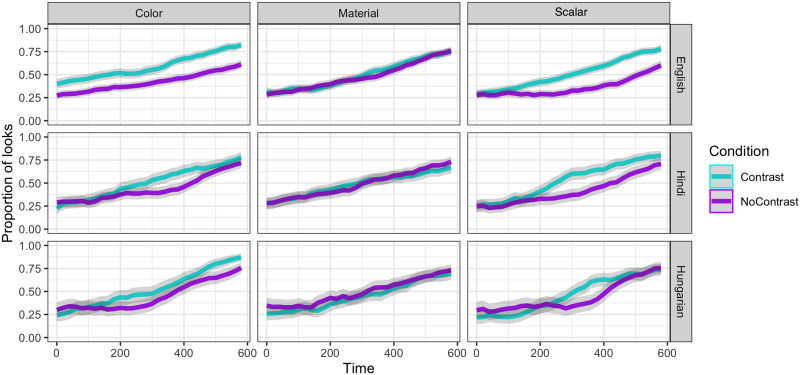
Proportion of looks to the target for each of the three languages in the critical time-window, which was time-locked to the onset of the noun. Grey ribbons represent 95% confidence intervals.

**Figure 3.**
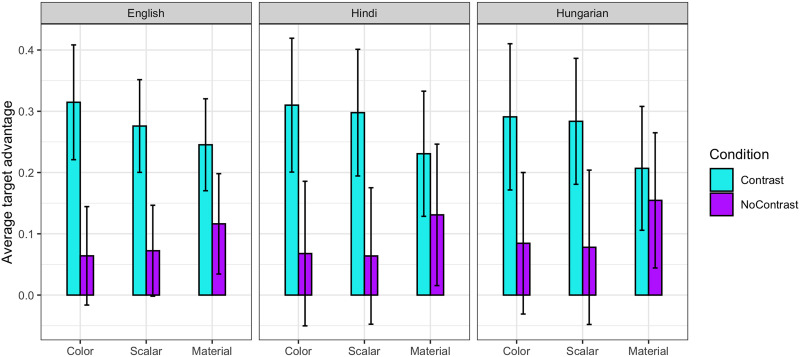
Target-advantage scores for each language and adjective type across the noun region. Error bars represent 95% confidence intervals.

Results are illustrated in Figures 2 and 3. The cluster-based permutation analyses showed a significantly different cluster of effects between conditions for color (from 240–600 ms, total sum of *t* values = 39.61, *p* < 0.01) and scalar adjectives (from 260–500 ms, total sum of *t* values = 33.07, *p* < 0.01) but not for material adjectives (no clusters whatsoever). There was also a significant cluster of effects for the interaction term (from 280–600 ms, total sum of *t* values = 37.96, *p* < 0.01).

The target-advantage score analysis showed a significant effect of CONDITION on the scores for color (*beta* = 0.24, *p* < 0.05, *t* = 2.41) and scalar adjectives (*beta* = 0.19, *p* < 0.05, *t* = 2.02), but not for those of material adjectives (*beta* = 0.10, *p* = 0.28, *t* = 1.08).

**Figure 4.**
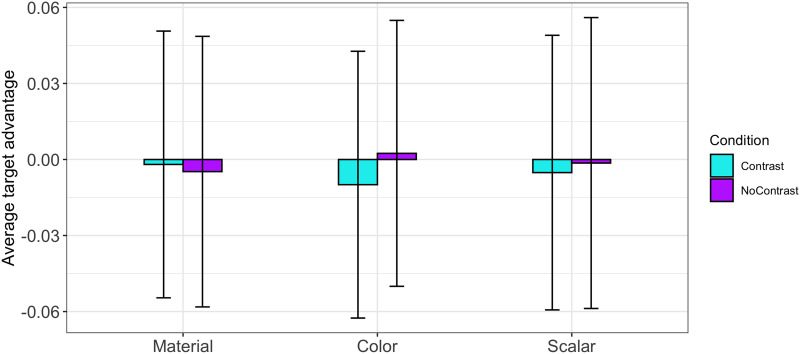
Target advantage scores prior to the critical time window (i.e., from trial onset to noun onset).

The target-advantage score model testing for baseline effects failed to find a difference between conditions (Contrast vs. No-Contrast). There was also no difference between adjectives (color, material, and scalar) and no interactions. Further, the grand mean (i.e., the intercept of the model) was not significantly different from zero, suggesting that, overall, there was no preference for either target or competitor prior to hearing the noun (see Figure 4). The results of this control analysis therefore confirm that the effects observed in the critical analyses were not due to the block design allowing participants to anticipate the target object. This also confirms that the filler trials—which were specifically designed to prevent participants from guessing the target—had the intended effect.

**Figure 5.**
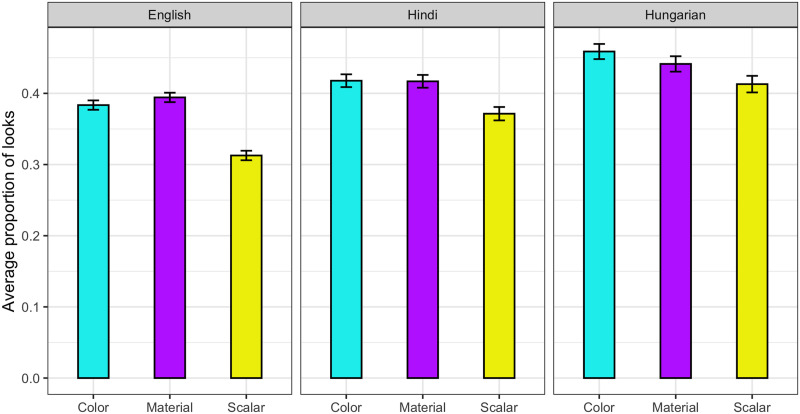
Proportion of looks to both target and competitor images in the No-Contrast condition for each of the three languages in the critical time-window, which was time-locked to the onset of the noun. Error bars represent 95% confidence intervals.

Finally, the analysis of overall looks to both target and competitor for each adjective type in the No-Contrast condition revealed significantly more looks to both images for color adjectives than for scalar adjectives (*beta* = 0.25, *p* < 0.01, *z* = 2.80). We also found a significant difference between material and scalar adjectives (*beta* = 0.24, *p* < 0.05, *z* = 2.40), suggesting that, overall, participants spent more time viewing the distractor images when hearing the scalar adjectives than when hearing color or material adjectives. These results can be seen in Figure 5.

## DISCUSSION

Here we used the classic eye-tracking paradigm by Sedivy et al. (1999; Sedivy, 2003, 2004) to test whether scalar, material and color adjectives elicit contrastive inferences in three languages with prenominal modification: English, Hindi, and Hungarian. Contrary to the original results in English (Sedivy, 2003, 2004) and in line with recent studies also using this paradigm in English (Aparicio, 2017; Aparicio et al., 2015; Saryazdi et al., 2022) or comparing English and Tsimane’ (Ryskin et al., 2023), we observed contrastive effects in the processing of color adjectives. That is, in hearing color descriptions such as ‘The black lamp,’ English, Hindi, and Hungarian speakers were faster to identify the target if there was a contrast object in the display (e.g., a yellow lamp), than if there was no contrast object (see Figure 1(2a–2b)).

These results challenge Sedivy’s (2003, 2004) argument that color adjectives do not elicit contrastive inferences because they are often used descriptively. While informativity expectations may indeed differ for different adjective types, recent reference production studies have shown that color adjectives tend to be used descriptively when it may help the listener’s visual search for the referent, but not across all visual contexts (Clarke et al., 2013; Gatt et al., 2017; Koolen et al., 2013; Long et al., 2021; Rubio-Fernandez, 2016, 2019, 2021; for modelling evidence, see Jara-Ettinger & Rubio-Fernandez, 2022). These studies therefore challenge the view that color adjectives are part of the default description of objects (Sedivy, 2003, 2004), suggesting instead that the communicative value of color adjectives may be higher than initially assumed. That is, the high discriminatory value of descriptive color adjectives may counter their low informativity value (for discussion, see Rubio-Fernandez et al., 2021).

Unlike color adjectives, and also contrary to the original findings (Sedivy, 2003, 2004), processing material adjectives did not elicit contrastive effects in our study. That is, in hearing ‘The leather shoes’, English, Hindi, and Hungarian speakers were not faster to identify the target in the Contrast condition (when there was a pair of canvas shoes in the display), than in the No-Contrast condition (see Figure 1(3a–3b)). We interpret these results as an effect of the low visual salience of material adjectives (Jara-Ettinger & Rubio-Fernandez, 2022; Kursat & Degen, 2021). As we argued in the Introduction, Sedivy’s eye-tracking paradigm requires that participants conceptualize the two objects of the same kind (e.g., two pairs of shoes) as contrasting along the relevant dimension (e.g., their material), in order to be able to anticipate the noun in hearing the adjective. However, because an object’s material is often not visually salient, participants may not readily perceive a material contrast in the display during the preview window, and therefore not be prepared to derive a contrastive inference in hearing a material adjective.

It must be noted, however, that the contrasting objects in the material trials also differed by color, which might have dampened the visual salience of the material contrast in comparison. In addition, the original eye-tracking studies by Sedivy (2003, 2004) employed physical objects instead of pictures, which might have increased the visual salience of material properties. Future eye-tracking studies using this paradigm should therefore employ pairs of physical objects that only vary along the relevant dimension (either color or material) for potentially stronger contrastive effects of material adjectives.

In summary, from a pragmatic perspective, color adjectives should elicit weaker contrast effects than material adjectives because color adjectives are often used descriptively (hence not warranting a contrastive interpretation), whereas material adjectives are rarely used non-contrastively (Sedivy, 2003, 2004). From a perceptual perspective, however, color adjectives should elicit greater contrast effects than material adjectives because color contrast is more visually salient than material contrast (Jara-Ettinger & Rubio-Fernandez, 2022; Kursat & Degen, 2021). The results of our study support the perceptual account rather than the informativity account, but it is important to note that both perceptual and pragmatic factors are closely related in a theory of efficient referential communication (Rubio-Fernandez, 2016, 2019; Rubio-Fernandez et al., 2021). Thus, as long as speakers are aware of the lower visual salience of an object’s material relative to its color, and use both types of adjectives when they have high discriminatory power in a given visual context (see Jara-Ettinger & Rubio-Fernandez, 2022; Long et al., 2021; Rubio-Fernandez, 2021), referential communication will be efficient.

Our findings also point to the influence of adjective semantics on gaze behavior. According to Aparicio et al.’s (2015; Aparicio, 2017) semantic analysis of scalar adjectives, color-matching objects should receive more fixations than scalar-matching objects because scalar adjectives are more context dependent and therefore potentially harder to process. However, these authors acknowledged that this effect might also be driven by color salience. Our results replicated Aparicio et al.’s (2015; Aparicio, 2017) results, revealing greater fixations to color-matching objects than to scalar-matching objects in the No-Contrast baseline across the three languages. Crucially, a significant difference in the same direction also emerged when comparing fixations to material-matching and scalar-matching objects. Because material is a less visually salient property than color, we interpret this difference as an effect of the different semantics of these adjectives—with scalar adjectives being more context dependent and hence harder to process than color and material adjectives in the absence of a contrast set, as predicted by Aparicio et al. (2015; Aparicio, 2017).

Finally, our results with scalar adjectives revealed contrastive effects across the three languages, replicating the original findings by Sedivy et al. (1999; Sedivy, 2004), as well as more recent eye-tracking studies using the same paradigm (Aparicio, 2017; Aparicio et al., 2015; Ryskin et al., 2023). Importantly, our results with color, material and scalar adjectives were similar regardless of the eye-tracking measure we employed (i.e., target fixation or target advantage), also in line with the results of Ryskin et al. (2023).

In conclusion, our results confirm that not only pragmatic (Sedivy, 2003, 2004) and semantic factors (Aparicio, 2017; Aparicio et al., 2015), but also perceptual factors (Jara-Ettinger & Rubio-Fernandez, 2022; Kursat & Degen, 2021) affect the derivation of contrastive inferences. The extent to which these multiple factors may affect the derivation of contrastive inferences depends not only on the adjective type but also on the visual context, which could explain the different results observed in this literature. In addition, the relative visual salience of different properties might be different for physical objects (Sedivy, 2003, 2004; Sedivy et al., 1999) than for pictures of objects presented on a computer screen (Aparicio, 2017; Aparicio et al., 2015; Rubio-Fernandez & Jara-Ettinger, 2020; Rubio-Fernandez et al., 2021; Ryskin et al., 2023; Saryazdi et al., 2022). Future eye-tracking studies using Sedivy’s classic paradigm should therefore employ physical objects for greater ecological validity and generalizability to everyday communication.

## FUNDING INFORMATION

This research was supported by a FRIPRO grant (#275505) from the Research Council of Norway awarded to PRF. CRR received support from the EyeHub network for eye-tracking research at the University of Oslo.

## AUTHOR CONTRIBUTIONS

C.R.R.: Data curation, Formal analysis, Validation, Visualization, Writing – original draft, Writing – review & editing. H.A.: Conceptualization, Validation, Writing – original draft, Writing – review & editing. M.L.: Investigation, Visualization. V.S.: Data curation, Formal analysis. J.J.-E.: Conceptualization, Data curation, Formal analysis, Validation. P.R.-F.: Conceptualization, Funding acquisition, Investigation, Methodology, Project administration, Resources, Supervision, Validation, Writing – original draft, Writing – review & editing.

## DATA AVAILABILITY STATEMENT

Data, analysis scripts and materials for this study are available in OSF. For the repository, see: https://osf.io/apxtj/?view_only=285b127c526b4ef58491ae3d963d3735.

## Note

^1^ Rubio-Fernandez and Jara-Ettinger (2020) did not investigate the effect of adjective type on the derivation of contrastive inferences. Therefore, the re-analysis of the Hindi and Hungarian data that we report here does not appear in the original paper.
